# Synthesis of Magnetic Wires from Polyol-Derived Fe-Glycolate Wires

**DOI:** 10.3390/nano10020318

**Published:** 2020-02-13

**Authors:** Shun Fujieda, Thomas Gaudisson, Jean-Marc Grenèche, Michel François, Souad Ammar

**Affiliations:** 1 Department of Industry and Technology, Graduate School of Engineering, Osaka University, Suita 565-0871, Japan; thomasgaudisson@gmail.com; 2 Institut des Molécules et Matériaux du Mans (IMMM), CNRS UMR-6283, Université du Maine, 72085 Le Mans Cedex, France; jean-marc.greneche@univ-lemans.fr; 3 Institut Jean Lamour (IJL), CNRS UMR-7198, Université de Lorraine, 54011 Nancy Cedex, France; michel.francois@univ-lorraine.fr; 4 Interfaces Traitement Organisation et Dynamique des Systèmes (ITODYS), CNRS UMR-7086, Université Paris, 75205 Paris Cedex, France; ammarmer@univ-paris-diderot.fr

**Keywords:** polyol process, magnetic wire, glycolate, iron oxide, iron metal

## Abstract

Fe-glycolate wires with micrometer-scale lengths can be synthesized by the polyol process. Although the as-produced wires are in the paramagnetic state at room temperature, they are transformed into ferrimagnetic iron oxides and ferromagnetic metallic iron wires by reductive annealing. The shape of the wires is unchanged by reductive annealing, and it is possible to control the magnetic properties of the resulting wire-shaped ferri/ferromagnets by adjusting the annealing conditions. Consequently, the reductive annealing of polyol-derived Fe-glycolate wires is an effective material-processing route for the production of magnetic wires.

## 1. Introduction

Chemical synthesis methods have been applied to the development of functional nanomaterials. Much effort has been devoted to controlling the morphology of the resulting materials, which is a key parameter to control properties or to induce new ones. In particular, wires have attracted much attention for their various applications: sensors [1], data storage [2], batteries [3], catalysis [4], biomedical [5], etc.

A reaction mediated by polyols, known as the polyol process, has been widely used to synthesize functional nanomaterials, such as metals, alloys, oxides, hydroxides and glycolates [6,7]. This process can be easily adapted to large-scale production, which is very important from a practical viewpoint [7]. In addition, a number of wires have been synthesized in this way. For example, Ag [8] and Cu [9] wires for transparent electrode applications have been synthesized using capping agents. The synthesis of Bi metal wires has also been reported [10]. Co and Co-Ni alloy wires for rare-earth-free magnet applications have been synthesized using nucleation agents [11,12].

Recently, we have now synthesized paramagnetic Fe-glycolate wires with micrometer-scale lengths by the polyol process. These wires consist of ferric ions complexed to chloride and diethylene glycolate ligands. Their transformation into magnetic iron metal and/or iron oxides under a reducing atmosphere is studied. By adjusting the reductive annealing conditions, we have produced magnetic wires.

## 2. Experiment

A diethylene glycol solution containing 5 mol/L of Fe(II) chloride was prepared. To obtain Fe-glycolate wires, the solution was heated to 160 ℃ and then maintained for 60 min. During the synthesis, any vapor was liquefied and returned to the solution. The precipitated Fe-glycolate wires were separated from the solution by centrifugation and washed repeatedly in ethanol. The recovered solids were then annealed under reductive conditions. Details are given in Table 1.

The transformation of the crystalline structure of these wires under a reducing atmosphere was studied by X-ray diffraction (XRD) measurements. The XRD patterns of the annealed samples were recorded at room temperature in ambient atmosphere, using Cu Kα radiation. The morphologies of the as-produced wires and their annealed counterparts were observed by scanning electron microscopy (SEM). They were also characterized by ^57^Fe Mössbauer spectrometry, using a conventional electromagnetic transducer with a triangular velocity form. A field emission-type electron microprobe analysis (EPMA) apparatus was used to analyze the chemical composition of the samples. Finally, the magnetic measurements were taken with a vibrating sample magnetometer (VSM).

## 3. Results and Discussion

**Figure 1:**
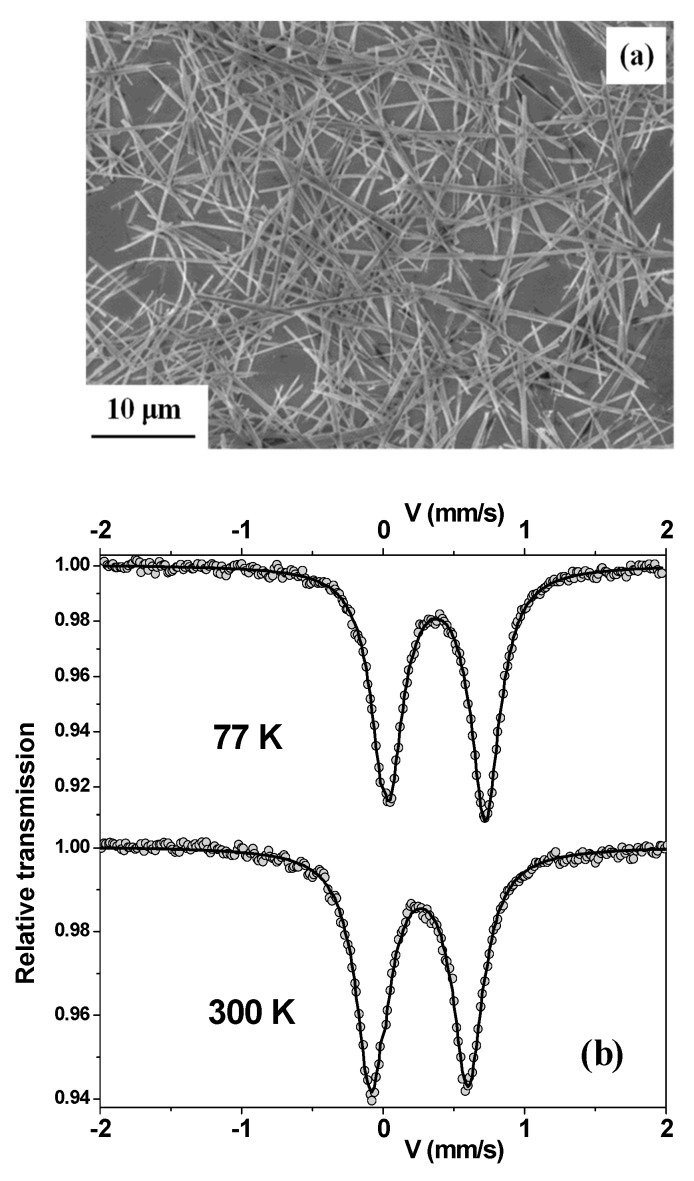
(**a**) Scanning electron microscopy (SEM) image of Fe-glycolate wires. (**b**) Mössbauer spectra at 77 and 300 K (recorded using magic-angle configuration).

Figure 1a shows an SEM image of the as-produced wires, which have diameters on the submicron scale and lengths of several tens of micrometers. Figure 1b indicates their Mössbauer spectra recorded at 77 and 300 K. At 77 K, the hyperfine structure is asymmetric and quadrupolar with narrow Lorentzian lines, which can be described by a single component (Figure 1b, top). Thus, the wires are paramagnetic above 77 K. The asymmetry is probably due to the preferred orientation of the wires, which is related to their linear morphology. This is confirmed by the change in the asymmetric shape in the 300 K spectrum (Figure 1b bottom), as usually observed in one-dimensional materials [13,14]. The refined value of the spectral linewidth (0.25 mm/s) indicates the excellent crystalline quality of the wires while those of the isomer shift (0.38 and 0.49 mm/s at 300 and 77 K, respectively) are fully consistent with octahedrally coordinated Fe^3+^ ions.

**Figure 2:**
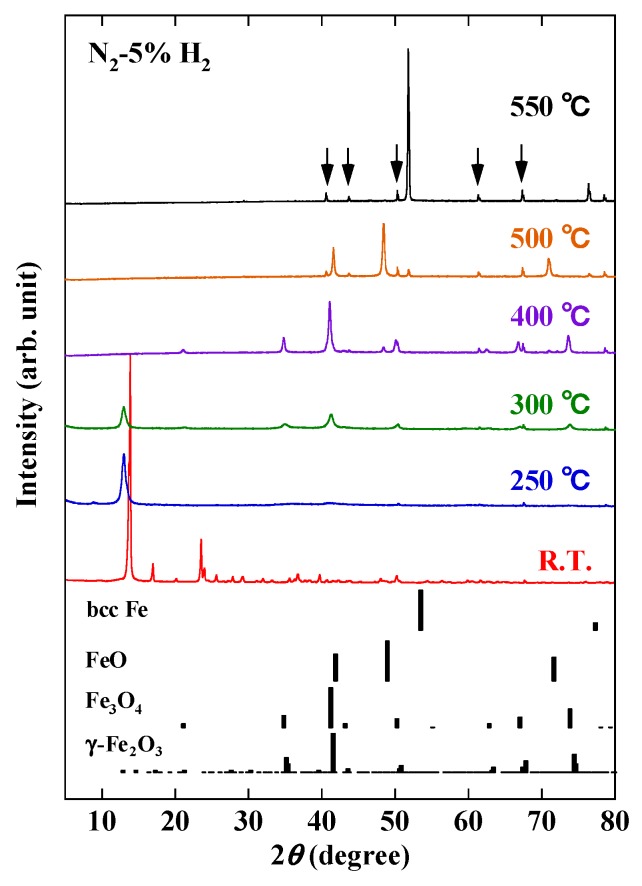
X-ray diffraction (XRD) patterns at various temperatures in N_2_-5% H_2_ atmosphere. Reference diffraction patterns for bcc Fe, FeO, Fe_3_O_4_ and γ -Fe_2_O_3_ are also given. Arrows in Figure indicate diffraction peaks due to sample holder.

To clarify the transformation of the crystalline structure of the wires under a reducing atmosphere (N_2_-5% H_2_ gas), the XRD patterns as a function of temperature were compared, together with tabulated ones for metallic iron (bcc Fe), wüstite (FeO), magnetite (Fe_3_O_4_) and maghemite (γ-Fe_2_O_3_) references (Figure 2). The arrows in the Figure indicate the diffraction peaks of the Al_2_O_3_ sample holder. The wires show clear diffraction peaks (see room temperature data), which were observed up to 200 °C. The resolution of the exact crystal structure of this phase will be published elsewhere. Briefly, it consists of Fe^3+^ ions octahedrally coordinated to one chlorine and five oxygen atoms, the latter being provided by three diethylene glycol molecules. The resulting octahedral Fe(O,Cl)_6_ units share edges and form zig-zag chains running along the *c*-axis of the crystal lattice. It has been reported that the addition of capping and nucleation agents to a polyol solution is necessary to synthesize wires (see, for example, works on the polyol-mediated synthesis of metallic Ag, Cu, Bi, Co and Co-Ni wires [8,9,10,11,12]). In the present case, Fe-glycolate wires were synthesized without the addition of similar agents. Therefore, their shape is associated with their nearly one-dimensional crystal structure.

The crystal structure transformation occurs between 200 and 250 °C (Figure 2). No diffraction peaks of a polyol-derived Fe-glycolate are found at 250 °C. The diffraction pattern at 250 °C has characteristics similar to those of a brucite-like layer structure [15]. Peaks of iron oxides, such as Fe_3_O_4_, consisting of Fe^2+^ and Fe^3+^ cations, and/or γ-Fe_2_O_3_, with only Fe^3+^ cations, are obtained at 300 °C. These diffraction peaks become clearer at 400 °C, although it is difficult to distinguish between Fe_3_O_4_ and γ-Fe_2_O_3_ under the experimental conditions used. Above 400 °C new peaks appear, indicating the transformation into FeO with only Fe^2+^. The peaks of FeO are clearly observed at 500 °C, accompanied by additional small peaks of bcc Fe. The reduction of Fe^2+^ into Fe^0^ seems to be complete at 550 °C, where the peaks of bcc Fe predominate. It is well established that, at room temperature, Fe_3_O_4_ and γ-Fe_2_O_3_ exhibit ferrimagnetic behavior while bcc Fe is ferromagnetic. Therefore, the magnetic functionality is obtained at room temperature by reductive annealing of the paramagnetic Fe-glycolate wires.

**Figure 3:**
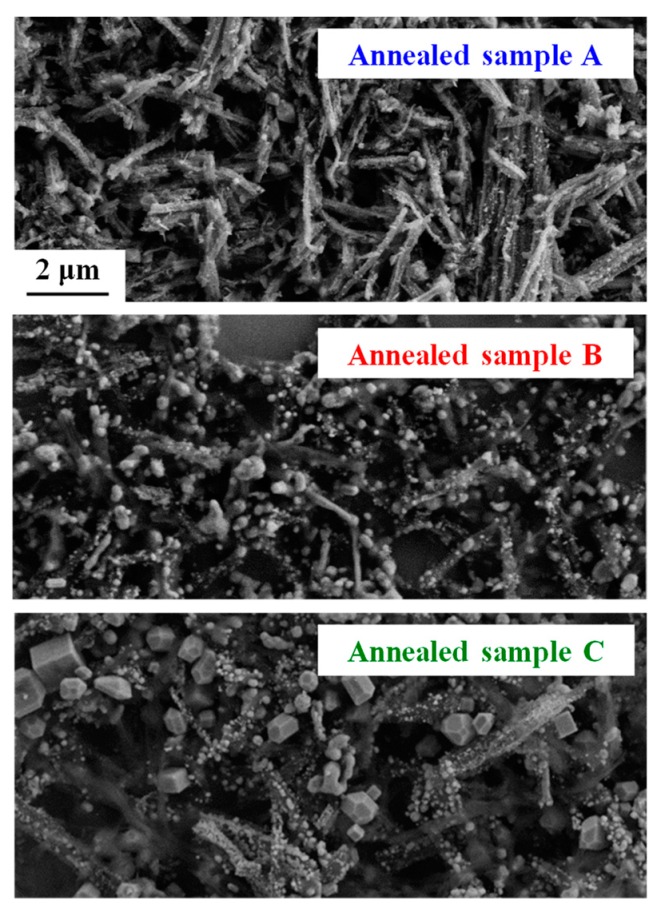
SEM images of annealed samples A, B and C. (the scale is the same for the 3 images)

The reduction reaction depends on the annealing conditions, such as hydrogen gas concentration, temperature and time. To study the influence of the conditions on morphology and magnetic properties, the wires were annealed under different operating conditions (Table 1). Figure 3 compares the SEM images of the annealed samples. It should be noted that the wire-like shape is retained in sample A, although the aspect ratio of the wires is reduced. Samples B and C have the same general shape, but small spherical particles are present in sample B and larger cubic particles are obtained in sample C.

**Figure 4:**
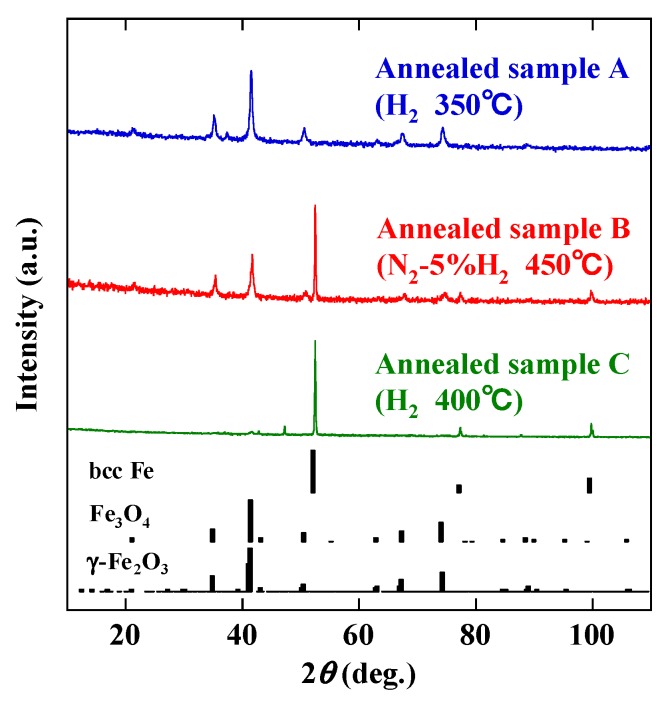
XRD patterns of annealed samples A, B and C, and reference patterns for bcc Fe, Fe_3_O_4_ and γ -Fe_2_O_3._

**Figure 5:**
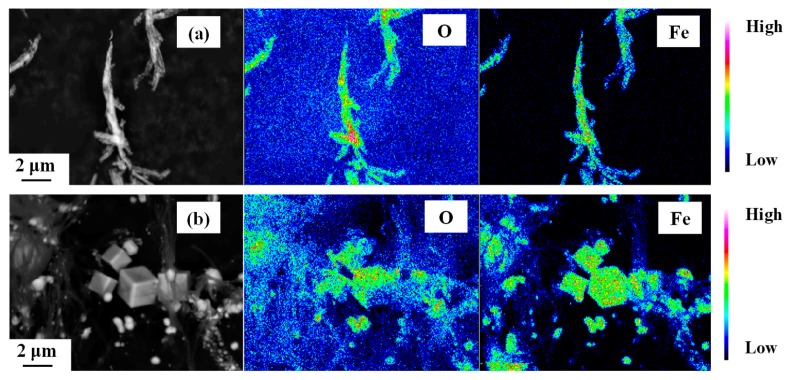
Backscatter electron images (**right**) and elemental maps obtained from field emission-type electron microprobe analysis at O-*K* (**center**) and Fe-*L* (**left**) edges of samples (**a**) A and (**b**) C.

Figure 4 shows the XRD patterns of these annealed samples, together with those tabulated for bcc Fe, Fe_3_O_4_ and γ-Fe_2_O_3_ references. Sample A is mainly consistent with Fe_3_O_4_ and/or γ-Fe_2_O_3_. On the other hand, sample C is mainly bcc Fe, while sample B is a mixture of iron oxides (Fe_3_O_4_ and/or γ-Fe_2_O_3_) and bcc Fe. The backscatter electron images and the elemental composition maps obtained from the characteristic Fe-*L* and O-*K* X-ray intensities of samples A and C are compared in Figure 5. The filamentary materials of sample A exhibit a relatively high intensity of Fe and O (Figure 5a). On the other hand, the cubic particles in sample C show a relatively high intensity of Fe and a lower intensity of O (Figure 5b). Therefore, it is clear that wire-like materials and cubic particles are mainly composed of iron oxides and iron metal, respectively. Sample B is probably a mixture of iron oxide wires and metallic iron particles.

**Figure 6:**
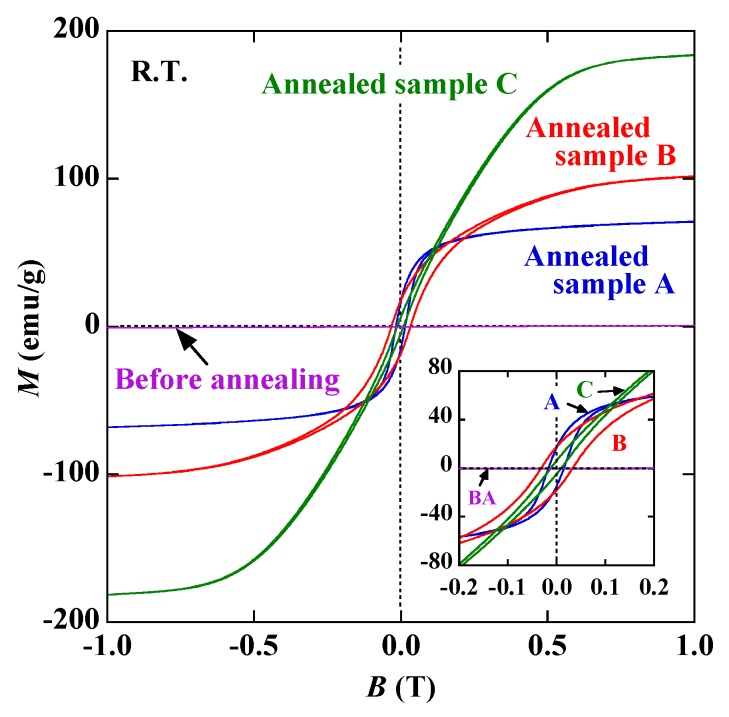
Room temperature magnetization curves of as-produced wires (before annealing (BA)) and samples A, B and C. Inset shows curves enlarged between −0.2 and 0.2 T.

Figure 6 compares the room temperature magnetization curves of samples A, B and C. The curve of the wires before annealing is also given. Whereas the as-produced wires are paramagnetic, all annealed samples are ferrimagnetic and/or ferromagnetic. The saturation magnetization *M*_s_ of bcc Fe is about 218 emu/g, which is much larger than those of Fe_3_O_4_ (96 emu/g) and γ-Fe_2_O_3_ (76 emu/g) [16]. Since sample C shows the highest bcc Fe content, the value of *M*_s_ is the largest. In contrast, sample A hardly contains any bcc Fe and has the smallest *M*_s_. Thus, the change in *M*_s_ can be explained qualitatively by the XRD results in Figure 4. The coercivity *H*_c_ is sensitive to the annealing conditions. The highest value of *H*_c_ is obtained for sample B, but it is difficult to determine its origin from the current data because coercivity is affected by various factors, such as crystal structure, crystallite size, sample size, impurities and magnetic interactions between the particles [17]. 

## 4. Conclusions

The transformation of the crystal structure of polyol-derived micrometer-long Fe-glycolate wires was studied by annealing in a reducing atmosphere. Although the wires are paramagnetic at room temperature, they are transformed into ferrimagnetic iron oxides, such as Fe_3_O_4_ and γ-Fe_2_O_3_, and ferromagnetic bcc Fe, when the annealing temperature is increased, but with no change in shape. Thus, magnetic wires were obtained by annealing the as-produced Fe-glycolate wires. Their magnetic properties were controlled by adjusting the annealing conditions. Consequently, the reductive annealing of polyol-derived Fe-glycolate wire is an effective process for obtaining magnetic wires.

## Figures and Tables

**Table 1:** Conditions for reductive annealing of polyol-derived Fe-glycolate wires.

Sample	Atmosphere	Temperature (°C)	Time (h)
A	H_2_	350	1
B	N_2_-5% H_2_	450	1
C	H_2_	400	1
